# Investigating the association between calcium–phosphorus balance and osteoarthritis: Evidence from NHANES 2007–2016

**DOI:** 10.1097/MD.0000000000043301

**Published:** 2025-07-18

**Authors:** Junjie Kang, Suyalatu Xin, Huxiong Zhang, Tiantian Wang, Keyu Zhao, Xiaoyu Gao, Yonghua Wang

**Affiliations:** a Department of Orthopedics Ward 1, Ordos Central Hospital, Ordos, Inner Mongolia, China.

**Keywords:** calcium–phosphorus balance, NHANES, osteoarthritis, serum calcium level, serum phosphorus level

## Abstract

The role of the calcium–phosphorus balance in influencing osteoarthritis (OA) risk is not fully understood. This study investigated the relationship between calcium–phosphorus metabolism and OA risk. Data from the National Health and Nutrition Examination Survey spanning 2007 to 2016 were used in this study. Participants aged ≥ 30 years with available serum calcium and phosphorus levels, self-reported OA status, and relevant covariates were included in the study. Multivariable logistic regression analysis was performed to assess the potential influence of calcium–phosphorus metabolism on the risk of OA. Nonlinear relationships were assessed using the restricted cubic spline model. Participants with OA had mildly elevated calcium and phosphorus levels. Concurrently, the calcium–phosphorus (Ca/P) ratio decreased. With confounders adjusted for, the Ca/P ratio was inversely associated with OA risk (odds ratio (OR) = 0.72, 95% CI: 0.56–0.94, *P* = .017), while serum calcium (OR = 2.12, 95% confidence interval (CI): 0.96–4.68, *P* = .064) and phosphorus (OR = 1.87, 95% CI: 1.22–2.86, *P* < .01) were positively associated. The relationship between the Ca/P ratio and risk of OA was significantly influenced by body mass index (*P* for interaction < .05). The restricted cubic spline model indicated that the Ca/P ratio was nonlinearly associated with OA risk with a threshold of 1.96. Serum calcium level was associated with OA in a *U*-shaped pattern, with a threshold of 2.35. In contrast, a linear association was observed between serum phosphorus levels. A reduced Ca/P ratio, increased serum phosphorus, and either elevated or decreased serum calcium each contribute to a heightened risk of OA. Imbalances in calcium–phosphorus metabolism may be associated with osteoarthritis risk. Relevant biomarkers, such as serum calcium, phosphorus, and the Ca/P ratio, could serve as potential indicators. Further studies are needed to confirm these findings.

## 1. Introduction

Osteoarthritis (OA) is a degenerative joint disorder characterized by progressive degradation of articular cartilage, resulting in pain, stiffness, and diminished range of motion, affecting hundreds of millions of people worldwide.^[1]^ In the United States (US), approximately 32.5 million adults are affected by this condition, accounting for 13% of the adult population.^[2]^ However, the incidence of OA is projected to rise annually, raising significant public health concerns as the population ages and obesity becomes more prevalent.^[3]^

Calcium-phosphorus metabolism plays a significant role in maintaining muscle coordination and the structural integrity of both bone and cartilage.^[4–6]^ The calcium–phosphorus (Ca/P) ratio, along with serum calcium and phosphorus levels, serves as a critical indicator of the calcium–phosphorus balance.^[7]^ These markers are not only important diagnostic indicators for osteoporosis, chronic kidney disease, and cardiovascular diseases but are also key prognostic markers for the progression and outcomes of these conditions.^[8,9]^ Imbalances in calcium–phosphorus impair muscle coordination, thereby disrupting the mechanical stress balance within the knee joint, which results in localized stress concentration and accelerates joint degeneration.^[10,11]^ Clinical research has indicated that patients with abnormal serum calcium or phosphorus levels experience more severe joint damage.^[12]^ Furthermore, an imbalance in the calcium–phosphorus balance is associated with more pronounced symptoms and joint dysfunction in osteoarthritic patients.^[13]^ Preclinical studies have sought to confirm this causality by demonstrating how this imbalance promotes inflammation. An imbalance in calcium–phosphorus metabolism can lead to the deposition of calcium and phosphate crystals within the joint, thereby activating synovial inflammation.^[14]^ The release of pro-inflammatory cytokines, including IL-1β, IL-6, IL-17, TNF-α, and chemokines, such as CXCL13, CCL8, and CCL5, from synovial tissue intensifies intra-articular inflammation.^[15,16]^ This inflammatory response activates matrix-degrading enzymes, including MMP-9 and MMP-13, thereby accelerating the degeneration of joint structures.^[17,18]^

Although previous studies have indicated that calcium–phosphorus imbalance is linked to the synovial inflammatory response and disease progression in OA patients, the association between calcium and phosphorus imbalance and the prevalence of OA remains ambiguous. Therefore, this study aimed to elucidate the association between calcium and phosphorus balance markers and the prevalence of OA. By investigating the association between distinct patterns of calcium–phosphorus imbalance and the prevalence of OA, we aimed to delineate the underlying mechanisms driving the pathophysiological process of OA and identify potential novel markers and therapeutic targets for prevention and intervention.

## 2. Material and methods

### 2.1. Study cohort

**Figure 1.**
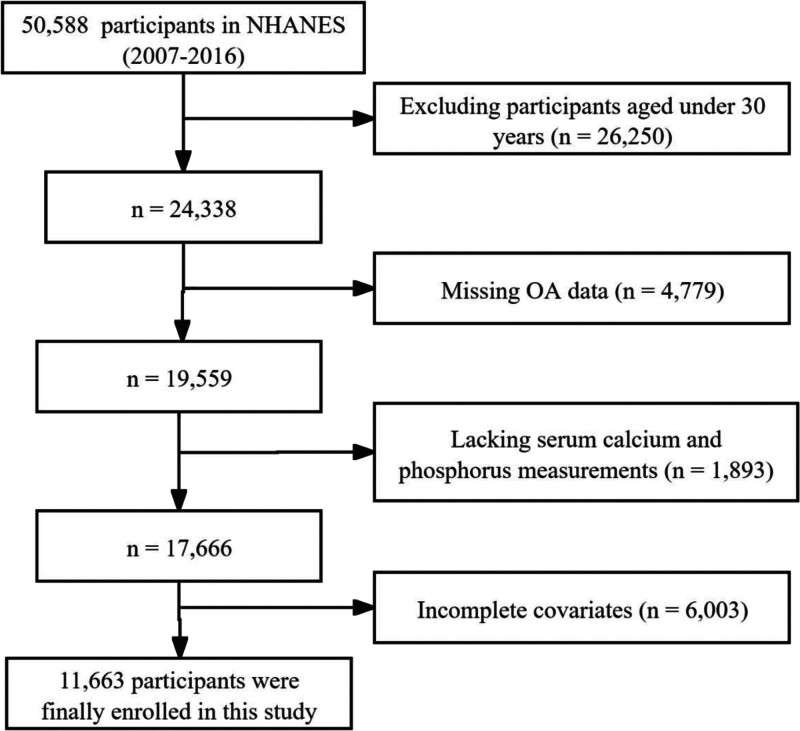
Flowchart of participants selection in this study.

In this study, data were collected from the 2006 to 2017 cycles of the National Health and Nutrition Examination Survey (NHANES), which uses a stratified, multi-stage sampling framework to ensure national representativeness.^[19]^ Figure 1 shows participant selection from the NHANES 2006 to 2017 cycles, excluding participants under 30 (n = 26,250), missing OA data (n = 4779), lacking serum calcium or phosphorus measurements (n = 1893), and with incomplete covariates (n = 6003), resulting in a final sample of 11,663 participants.

### 2.2. OA and Non-OA classification

The classification of participants as having or without OA was determined through structured questionnaire interviews. Participants were initially asked, “Has a doctor ever diagnosed you with arthritis?” A “yes” answer prompted the following inquiry: “What specific form of arthritis was diagnosed?” Participants who responded “yes” to the initial question and were informed “OA” in the second question were classified as having OA. Conversely, those who responded ‘no’ to the initial question were categorized as non-OA.

### 2.3. Calcium levels, phosphorus levels and the Ca/P ratio

Blood samples obtained from the mobile examination center were analyzed to measure calcium and phosphorus concentrations as part of standard biochemical testing. The Beckman UniCel DxC 800 Synchron Clinical System was used, employing an indirect ion-selective electrode method for calcium and a time rate method for phosphorus. The Ca/P ratio was calculated by dividing serum calcium by the phosphorus concentration.

### 2.4. Assessment of covariates

Several essential covariates were selected based on the prior literature and their potential relevance to OA, in order to control for confounding effects.^[20–24]^ These included demographic variables (age, sex, race, and education), body mass index (BMI), lifestyle characteristics (smoking status, alcohol consumption, and physical activity), and the presence of diabetes. Participants were divided into 3 distinct groups according to their BMI: underweight/normal (<25), overweight (25 < 30), and obese (≥30). Smoking status was classified as never smoker (having smoked fewer than 100 cigarettes in their lifetime), current smoker (having smoked at least 100 cigarettes and still smoking), or former smoker (having smoked at least 100 cigarettes but had quit). Alcohol consumption was categorized as nondrinkers (fewer than 12 drinks in their lifetime) or drinkers (<5, 5 to 10, or more than 10 drinks/month). The metabolic equivalent of task (MET) was calculated following the guidelines of the International Physical Activity Questionnaire.^[25]^ Total MET-min/wk were derived by summing the weekly minutes spent in vigorous and moderate physical activity or work: Total MET = (Vigorous min/wk × 8.0) + (Moderate min/wk × 4.0). According to the International Physical Activity Questionnaire scoring protocol, physical activity levels were classified into 3 categories: low activity (≤600 MET-min/wk), moderate activity (>600–≤3000 MET-min/wk), and high activity (>3000 MET-min/wk). Diabetes criteria included self-reported history, insulin use, fasting glucose level ≥7 mmol/L, and glycohemoglobin level ≥6.5%.

### 2.5. Statistical analysis

Considering the sophisticated sampling design of the NHANES and ensuring US population representativeness, all analyses accounted for sample weights, clustering, and stratification, and were conducted using R version 4.4.1. For baseline characteristics, continuous variables were summarized as mean with standard deviation, and categorical variables were described using counts and proportions. Student *t* test was applied to continuous variables, while the chi-square test was used for categorical variables. To assess potential selection bias, we compared the baseline characteristics of included and excluded participants. Multicollinearity among calcium levels, phosphorus levels, the Ca/P ratio, and the covariates was assessed using the generalized variance inflation factor (GVIF), following the method proposed by Fox and Monette.^[26]^ Logistic regression models were employed to assess the associations between calcium and phosphorus levels, the Ca/P ratio, and OA. Model 1 was analyzed without adjustments. Subsequently, Model 2 considers age and sex for adjustment. Finally, Model 3 was further adjusted by incorporating age, sex, race, education, BMI, MET, smoking status, alcohol consumption, and diabetes. Potential nonlinear associations were assessed using restricted cubic spline analysis, and segmented linear regression was applied to identify corresponding inflection points. Stratified analyses were performed to determine the consistency among subgroups.

## 3. Results

### 3.1. Baseline characteristics of participants with and without OA

The baseline characteristics of the 11,663 participants enrolled in this study are listed in Table 1. Participants with OA were older, predominantly female, had a higher BMI, were more likely to be former smokers, had a higher proportion of non-Hispanic White persons, had slightly higher education levels, lower MET, and a higher prevalence of diabetes (*P* < .05). In addition, participants with OA showed higher serum calcium and phosphorus levels but a lower Ca/P ratio (*P* < .05). Additionally, the characteristics of 11,663 included and 12,675 excluded participants were also compared (presented in Table S1, Supplemental Digital Content, https://links.lww.com/MD/P405). Included participants were younger, more male, Non-Hispanic White persons, and highly educated. They had lower BMI, high proportions of never smokers and individuals consuming >10 drinks/month, and lower diabetes prevalence (*P* < .05). Additionally, these participants had slightly higher levels of serum phosphorus and a lower Ca/P ratio (*P* < .05). However, there was no significant difference in serum calcium levels between the 2 groups. This result indicated that included participants may not be fully representative of the overall population.

**Table 1:** Baseline characteristics of individuals with and without OA.

Characteristic	Overall (n = 11,663)*	Non-OA (n = 10,100)*	OA (n = 1563)*	*P* †
Age (yr)	50.24 (13.48)	48.32 (12.79)	61.07 (12.06)	<.001
Gender (weighted %)				<.001
Female	5372 (47.56)	4425 (45.11)	947 (61.35)	
Male	6291 (52.44)	5675 (54.89)	616 (38.65)	
Race (weighted %)				<.001
Mexican American	1691 (7.33)	1586 (8.24)	105 (2.19)	
Other hispanic	1214 (4.89)	1099 (5.36)	115 (2.23)	
Non-hispanic White	5318 (71.78)	4307 (69.43)	1011 (84.96)	
Non-hispanic Black	2163 (9.11)	1938 (9.73)	225 (5.60)	
Other/multiracial	1277 (6.90)	1170 (7.24)	107 (5.01)	
Education (weighted %)				.011
<9th grade	1026 (4.23)	937 (4.51)	89 (2.67)	
9–11th grade (includes 12th grade with no diploma)	1436 (8.96)	1273 (9.17)	163 (7.77)	
High school graduate/GED or equivalent	2514 (20.48)	2177 (20.66)	337 (19.48)	
Some college or AA degree	3300 (29.93)	2803 (29.40)	497 (32.97)	
College graduate or above	3387 (36.39)	2910 (36.27)	477 (37.12)	
Body mass index (kg/m²)	28.61 (6.17)	28.40 (6.01)	29.77 (6.89)	<.001
Smoking status (weighted %)				<.001
Current smoker	2253 (17.81)	1996 (18.23)	257 (15.43)	
Former smoker	2992 (26.71)	2447 (25.10)	545 (35.76)	
Never smoker	6418 (55.48)	5657 (56.67)	761 (48.81)	
Alcohol consumption (weighted %)				.151
Nondrinker	5782 (49.18)	5017 (49.15)	765 (49.34)	
<5 drinks/mo	1966 (21.01)	1683 (20.96)	283 (21.28)	
5–10 drinks/mo	950 (9.63)	851 (9.99)	99 (7.65)	
>10 drinks/mo	2965 (20.18)	2549 (19.90)	416 (21.73)	
Diabetes (weighted %)				<.001
Diabetes	1746 (11.07)	1430 (10.15)	316 (16.24)	
Non-diabetes	9917 (88.93)	8670 (89.85)	1247 (83.76)	
MET (min/wk)	4356.21 (6017.60)	4537.34 (6228.41)	3339.23 (4529.01)	<.001
Calcium (mmol/L)	2.35 (0.09)	2.35 (0.09)	2.36 (0.10)	.002
Phosphorus (mmol/L)	1.20 (0.18)	1.20 (0.18)	1.23 (0.17)	<.001
Ca/P ratio	2.00 (0.31)	2.00 (0.31)	1.96 (0.29)	<.001

Ca/P = calcium–phosphorus, MET = metabolic equivalent of task, OA = osteoarthritis.

* Mean (SD); n (unweighted); % (weighted).

† Design-based *t* test; Pearson’s *X*^2^: Rao & Scott adjustment.

### 3.2. Association between biomarkers of calcium–phosphorus metabolism and OA

To investigate the relationship between the Ca/P ratio, calcium levels, phosphorus levels, and OA risk, 3 models were constructed. For the Ca/P ratio (Table 2), the odds ratio (OR) in Model 1 was 0.64 (95% CI: 0.50–0.80, *P* < .001). This indicates that the risk of OA is reduced with each unit increase in the ratio. This association remained significant in Model 2 (OR = 0.76, 95% CI: 0.57–0.97, *P* = .028) and Model 3 (OR = 0.72, 95% CI: 0.56–0.94, *P* = .017). The ORs for Q2, Q3, and Q4 of the ratio were 0.80 (95% CI: 0.66–0.96, *P* = .022), 0.82 (95% CI: 0.68–0.99, *P* = .044), and 0.77 (95% CI: 0.61–0.96, *P* = .024), with P for trend significant across all models (*P* < .001, .044, .028).

**Table 2:** Association of Ca/P ratio and OA.

	Model 1		Model 2		Model 3	
	OR (95% CI)	*P*	OR (95% CI)	*P*	OR (95% CI)	*P*
Ca/P ratio	0.64 (0.50–0.80)	<.001	0.76 (0.57–0.97)	.028	0.72 (0.56–0.94)	.017
Q1	References	–	References	–	References	–
Q2	0.81 (0.67–0.97)	.024	0.81 (0.67–0.98)	.037	0.80 (0.66–0.96)	.022
Q3	0.80 (0.67–0.95)	.015	0.83 (0.69–1.00)	.057	0.82 (0.68–0.99)	.044
Q4	0.69 (0.57–0.84)	<.001	0.79 (0.63–0.98)	.038	0.77 (0.61–0.96)	.024
*P* for trend	<.001		.044		.028	

Model 1: Unadjusted; no variables included. Model 2: Adjusted for age and gender. Model 3: Adjusted for age, gender, race, education, BMI, smoking status, alcohol consumption, MET and diabetes.

CI = confidence interval, OR = odds ratio.

Additionally, for calcium levels (Table 3), the OR in Model 1 was 3.84 (95% CI: 1.70–8.66, *P* = .001). However, it became nonsignificant in Model 2 (OR = 1.62, 95% CI: 0.73–3.57, *P* = .230) and remained near-significant in Model 3 (OR = 2.12, 95% CI: 0.96–4.68, *P* = .064), suggesting a potential positive association despite not reaching the conventional threshold for statistical significance. For Q2, Q3, and Q4 of the levels, the associated ORs were 0.94 (95% CI: 0.79–1.11, *P* = .438), 1.01 (95% CI: 0.80–1.28, *P* = .917), and 1.35 (95% CI: 1.13–1.62, *P* = .002), respectively. The significance of the *P* for trend was observed in model 1 (*P* = .005). However, it was not significant in model 2 (*P* = .167) and remained close to significance in model 3 (*P* = .042).

**Table 3:** Association between serum calcium levels concentration and OA.

	Model 1		Model 2		Model 3	
	OR (95% CI)	*P*	OR (95% CI)	*P*	OR (95% CI)	*P*
Calcium	3.84 (1.70–8.66)	.001	1.62 (0.73–3.57)	.230	2.12 (0.96–4.68)	.064
Q1	References	–	References	–	References	–
Q2	0.94 (0.79–1.11)	.438	0.90 (0.75–1.09)	.284	0.90 (0.74–1.09)	.280
Q3	1.01 (0.80–1.28)	.917	0.96 (0.74–1.25)	.770	0.99 (0.76–1.30)	.967
Q4	1.35 (1.13–1.62)	.002	1.16 (0.96–1.14)	.135	1.24 (1.03–1.50)	.029
*P* for trend	.005		.167		.042	

Model 1: Unadjusted; no variables included. Model 2: Adjusted for age and gender. Model 3: Adjusted for age, gender, race, education, BMI, smoking status, alcohol consumption, MET and diabetes.

CI = confidence interval, OR = odds ratio.

Furthermore, for phosphorus levels (Table 4), the OR in Model 1 was 2.36 (95% CI: 1.63–3.42, *P* < .001), indicating that higher phosphorus levels increased the risk of OA. The significance of this association was maintained in Model 2 (OR = 1.71, 95% CI: 1.12–2.63, *P* = .014) and Model 3 (OR = 1.87, 95% CI: 1.22–2.86, *P* = .005). The ORs for Q2, Q3, Q4 of the levels were 1.28 (95% CI: 1.05–1.56, *P* = .018), 1.27 (95% CI: 1.02–1.58, *P* = .033), and 1.63 (95% CI: 1.32–2.00, *P* < .001). The *P* value for trend was significant across all models (*P* < .001, .027, and .011).

**Table 4:** Association between serum phosphorus levels concentration and OA.

	Model 1		Model 2		Model 3	
	OR (95% CI)	*P*	OR (95% CI)	*P*	OR (95% CI)	*P*
Phosphorus	2.36 (1.63–3.42)	<.001	1.71 (1.12–2.63)	.014	1.87 (1.22–2.86)	.005
Q1	References	–	References	–	References	–
Q2	1.28 (1.05–1.56)	.018	1.14 (0.92–1.40)	.238	1.17 (0.94–1.44)	.163
Q3	1.27 (1.02–1.58)	.033	1.05 (0.82–1.34)	.714	1.08 (0.85–1.38)	.547
Q4	1.63 (1.32–2.00)	<.001	1.36 (1.07–1.72)	.013	1.42 (1.12–1.79)	.005
*P* for trend	<.001		.027		.011	

Model 1: Unadjusted; no variables included. Model 2: Adjusted for age and gender. Model 3: Adjusted for age, gender, race, education, BMI, smoking status, alcohol consumption, MET and diabetes.

CI = confidence interval, OR = odds ratio.

To evaluate potential multicollinearity among the covariates, we conducted a GVIF analysis. The results indicated that all covariates had GVIF values <5 and adjusted GVIF values <2 across all 3 models, suggesting no significant multicollinearity. Detailed GVIF diagnostics for the calcium level, phosphorus level, and Ca/P ratio models are presented in Tables S2–S4, Supplemental Digital Content, https://links.lww.com/MD/P405.

### 3.3. Nonlinear and threshold effects of calcium–phosphorus balance on osteoarthritis risk

**Figure 2.**
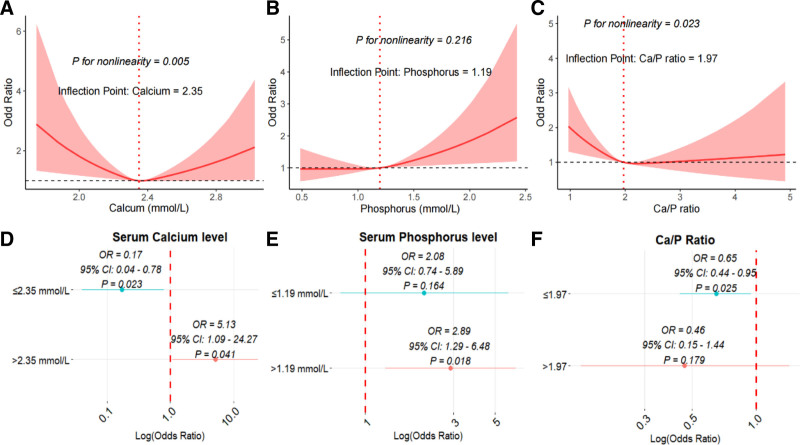
RCS curves and inflection points of calcium level (A), phosphorus level (B), and Ca/P ratio (C) with OA, with models adjusted for all covariates (e.g., age, gender, education and BMI). Threshold effect analysis of calcium level (D), phosphorus level (E), and Ca/P ratio (F) with OA risk. BMI = body mass index, Ca/P = calcium–phosphorus, OA = osteoarthritis.

We observed that serum levels were nonlinearly associated with OA risk, with an inflection point identified at 2.35 mmol/L (Fig. 2A; *P* for nonlinearity < .05). Among individuals with serum calcium levels <2.35 mmol/L, each unit increase in calcium level was associated with an 83% reduction in the odds of OA (Fig. 2D; OR = 0.17, 95% CI: 0.04–0.78, *P* = .023). Conversely, for individuals with serum calcium levels >2.35 mmol/L, each unit increase in calcium level was associated with a significantly increased risk of OA (OR = 5.13, 95% CI: 1.09–24.27, *P* = .041). Serum phosphorus levels exhibited a linear association with OA risk, with an inflection point identified at 1.19 mmol/L (Fig. 2B; *P* for nonlinearity = .216). Individuals with serum phosphorus levels above this threshold had a significantly increased risk of developing OA (Fig. 2E; OR = 2.89, 95% CI: 1.29–6.48, *P* = .018). Furthermore, the Ca/P ratio demonstrated a nonlinear association with OA risk, with an inflection point identified at 1.97 (Fig. 2C, *P* for nonlinearity < .05). For individuals with Ca/P ratios below 1.97, each unit increase was associated with a 35% reduction in the odds of OA (Fig. 2F; OR = 0.65, 95% CI: 0.44–0.95, *P* = .025). However, for those with a Ca/P ratio above this threshold, the association with OA risk was not statistically significant (OR = 0.46, 95% CI: 0.15–1.44, *P* = .179).

### 3.4. Subgroup analysis of calcium–phosphorus balance and osteoarthritis risk

Subgroup analyses were conducted based on demographic variables, BMI, lifestyle characteristics, and diabetes to investigate the relationship between Ca/P ratio and OA risk (Table 5). The negative association was more pronounced among overweight individuals (25 ≤ BMI < 30), never-smokers, those with high physical activity levels, and non-diabetics (*P* < .01). Additionally, with the exception of BMI (*P* for interaction < .05), none of the above characteristics showed a significant interaction with OA risk (*P* for interaction > .05).

**Table 5:** Association of Ca/P ratio and OA between different subgroups.

Subgroup	OR	95% CI	*P*	*P* for interaction
Age (yr)				**.583**
30–39	0.83	0.30–2.30	.715	
40–49	0.68	0.38–1.23	.197	
50–59	0.91	0.57–1.46	.704	
60–69	0.60	0.34–1.06	.080	
70–79	0.64	0.33–1.24	.184	
80+	1.05	1.45–2.46	.912	
Gender				**.114**
Female	0.97	0.66–1.42	.870	
Male	0.62	0.42–0.93	.021	
Race				**.789**
Mexican American	1.51	0.58–3.94	.388	
Other hispanic	0.49	0.19–1.26	.137	
Non-hispanic White	0.72	0.54–0.98	.035	
Non-hispanic Black	0.66	0.35–1.24	.192	
Other/multiracial	1.01	0.33–3.28	.977	
Education				**.092**
<9th grade	0.41	0.09–1.79	.234	
9–11th grade (includes 12th grade with no diploma)	0.29	0.11–0.78	.016	
High school graduate/GED or equivalent	1.04	0.63–1.74	.870	
Some college or AA degree	1.11	0.19–1.79	.635	
College graduate or above	0.52	0.32–0.86	.012	
Body mass index (kg/m²)				**.031**
Underweight & normal	0.92	0.52–1.61	.755	
Overweight	0.41	0.24–0.69	.001	
Obesity	1.05	0.72–1.54	.789	
Smoking status				**.568**
Current smoker	0.80	0.48–1.33	.380	
Former smoker	0.99	0.57–1.74	.988	
Never smoker	0.59	0.42–0.82	.002	
Alcohol consumption				**.680**
Nondrinker	1.07	0.62–1.84	.806	
<5 drinks/mo	0.74	0.51–1.07	.106	
5–10 drinks/mo	0.56	0.17–1.83	.333	
>10 drinks/mo	0.55	0.30–0.99	.048	
Physical activity level				**.120**
Low activity	1.07	0.64–1.80	.795	
Moderate activity	0.76	0.51–1.15	.188	
High activity	0.55	0.37–0.80	.002	
Diabetes				**.573**
Diabetes	1.05	0.57–1.95	.872	
Non-diabetes	0.68	0.51–0.90	.007	

Low activity, ≤600 MET-min/wk; moderate activity, 600 < MET-min/wk ≤3000; high activity, >3000 MET-min/wk.

Adjusted for age, gender, race/ethnicity, education, BMI, smoking status, alcohol consumption, physical activity, and diabetes, excluding the subgroup variable analyzed.

*P*‐values in bold are intended to enhance visual distinction between subgroup analysis *P*‐values and interaction test *P*‐values.

Ca/P = calcium–phosphorus, MET = metabolic equivalent of task, OA = osteoarthritis.

## 4. Discussion

In this large-scale cross-sectional study, we observed that individuals with OA exhibited higher serum calcium and phosphorus levels, as well as a lower Ca/P ratio. Additionally, we identified a significant correlation between biomarkers of calcium–phosphorus balance and the prevalence of OA. Specifically, we observed a negative correlation between the Ca/P ratio and OA risk, a positive association between serum phosphorus levels and OA risk, and a U-shaped relationship between serum calcium levels, with both high and low calcium levels associated with increased OA risk. Our results suggest that calcium–phosphorus imbalance may be associated with an increased risk of OA, and managing the calcium–phosphorus metabolic balance could potentially mitigate the occurrence of OA.

As population aging intensifies, the prevalence of OA has steadily increased and is emerging as a major public health issue.^[27]^ Recent studies have demonstrated that calcium–phosphorus metabolic imbalances adversely affect the homeostasis of bone and cartilage.^[28,29]^ Therefore, it is essential to identify biomarkers associated with the calcium–phosphorus metabolic balance and the risk of OA.

Our study found that serum calcium levels above or below 2.35 mmol/L, serum phosphorus levels above 1.19 mmol/L, and the Ca/P ratio below 1.96 were all associated with an increased risk of OA. For adults, the normal reference range for total serum calcium is 2.12 to 2.62 mmol/L, and for serum phosphorus, it is 0.81 to 1.45 mmol/L.^[30]^ However, there is no universally accepted reference range for the Ca/P ratio. Reduction in the Ca/P ratio reflects an underlying disturbance in mineral metabolism, commonly due to increased phosphorus or reduced calcium concentrations.^[31]^ Serum phosphorus, predominantly present as inorganic phosphate, is primarily derived from dietary intake and phosphate additives, with consumption in the US consistently exceeding established dietary recommendations.^[32]^ Excessive phosphorus intake contributes to an increased systemic phosphorus burden, resulting in phosphate accumulation within chondrocytes and promoting cartilage calcification.^[33]^ Elevated calcium and phosphate levels within the cartilage microenvironment further facilitate the formation of calcium–phosphate (Ca–Pi) complexes. These Ca–Pi complexes, primarily composed of basic calcium phosphate (BCP) and calcium pyrophosphate dihydrate (CPPD) crystals, can activate the NF-κB, p38, and ERK1/2 signaling pathways, thereby promoting the expression of MMP-3 and MMP-13, which contribute to early cartilage degeneration and extracellular matrix degradation.^[34]^ A case-control study demonstrated that OA patients in the Unite State exhibited higher levels of phosphorus intake, suggesting that excessive phosphorus intake may be a risk factor for OA.^[35]^ In vitro experiment, Duan et al reported that low phosphorus intake can alleviate nucleic acid damage induced cartilage calcification and reduce the local accumulation of inorganic phosphate.^[36]^ Additionally, reduction in the Ca/P ratio has been associated with decreased serum calcium levels. Insufficient calcium levels have been shown to prompt hyperparathyroidism (HPT), which leads to stimulates local bone remodeling.^[37]^ In response to elevated parathyroid hormone (PTH) levels, local calcium concentrations within the joint may become elevated, which may result in the deposition of Ca–Pi complexes.^[38]^ Reduced serum calcium levels may also disrupt calcium-dependent signaling pathways in chondrocytes, resulting in decreased production of type II collagen.^[39]^ A study conducted by Tudorachi et al, which included 326 individuals with OA, reported that serum calcium levels were negatively associated with radiographic progression, indicating that lower calcium levels may be related to the progression of OA.^[40]^ Furthermore, serum calcium insufficient is generally consider associated with Vitamin D deficiency. Vitamin D deficiency promotes the production of IL-1β, IL-10, and TNF-α by activating the NF-κB signaling pathway, thereby triggering inflammatory responses and accelerating chondrocyte degradation.^[41]^ These are all crucial factors in the degeneration of osteoarthritis cartilage. Several reasons can be put forward to explain why the elevated serum calcium raises the risk of OA. Elevated serum calcium may prompt the cartilage calcification, a pathological process implicated in the development of OA.^[42]^ In addition, active bone remodeling is often reflected by elevated serum calcium levels, which may accelerate subchondral sclerosis and trabecular disruption, and contribute to the deposition of BCP and CPPD crystals.^[43,44]^ These crystals may be phagocytosed by synovial macrophages, leading to activation of the NLRP3 inflammasome and subsequent release of pro-inflammatory cytokines such as IL-1β and TNF-α, which in turn induce chondrocyte and extracellular matrix degradation.^[45]^ Furthermore, elevated serum calcium levels are commonly associated with HPT.^[46]^ A cross-sectional study conducted among Korean women reported that those in the highest quartile of PTH levels exhibited a significantly increased prevalence of OA.^[47]^ Among patients with HPT, deposition of BCP and CPPD crystals in the synovium has been observed, which can promote local inflammation and cartilage degeneration.^[48]^

OA is a multifactorial disease characterized by cartilage and subchondral bone degradation, with low-grade chronic inflammation.^[49]^ Although the pathogenesis of OA remains unclear, the interaction between calcium–phosphorus metabolism and the musculoskeletal system plays a critical role in its development.^[50]^ Calcium-phosphorus metabolism is involved in regulating the stress balance of the musculoskeletal system and maintaining bone strength.^[51]^ Calcium-phosphorus metabolic imbalance disrupts muscle coordination, causing a stress imbalance that accelerates cartilage degradation and subchondral bone remodeling, ultimately accelerating joint degeneration.^[52,53]^ Additionally, calcium–phosphorus imbalance disrupts phosphate metabolism and promotes CPPD deposition and synovial inflammation, both of which are risk factors for OA.^[54,55]^ This imbalance also affects chondrocyte proliferation and differentiation as well as alterations in subchondral bone, which are considered early signs of OA.^[56,57]^ However, the exact role of the calcium–phosphorus balance in the pathogenesis of OA remains inadequately understood, warranting additional studies to elucidate the complex relationship between calcium–phosphorus metabolism imbalance and OA.

Our study has several strengths. First, it utilized a large representative cohort of 11,663 participants, which ensured the robustness and generalizability of the results. Second, by adjusting for key covariates and controlling for confounders, we identified biomarkers of calcium–phosphorus metabolism balance that are independently associated with OA risk. Moreover, serum calcium and phosphorus levels and the Ca/P ratio serve as cost-effective laboratory examinations for assessing calcium–phosphorus metabolism balance. It is valuable for screening and intervention in high-risk populations as well as for data collection in large-scale cohort studies.

Nevertheless, this study had certain limitations. First, the data were derived from the NHANES database, which reflects population trends in the Unite State. Therefore, these findings may not be directly applicable to populations from other regions. Furthermore, despite the large sample size, the evaluation of biomarkers related to calcium–phosphorus balance in our study primarily relied on single-point laboratory measurements. This could introduce a degree of potentially influence the results. Another limitation is that the classification of OA primarily relies on participants’ self-reported physician diagnosis, which may lead to an underestimation of individuals with asymptomatic OA. Notably, the difference between the included and excluded participants means that the result of our findings may be limited in its representativeness for all populations. Given that it is a cross-sectional study, it is insufficient to determine the causal correlation. Consequently, additional longitudinal studies and repeated assessments are essential to offer more robust and reliable insights into the association between OA and biomarkers of calcium–phosphorus metabolism balance.

## 5. Conclusions

Our study suggests that imbalances in calcium–phosphorus metabolism may be associated with the risk of OA. Biomarkers such as serum calcium, serum phosphorus, and the Ca/P ratio may serve as potential laboratory indicators for this association. These findings highlight the potential value of assessing calcium–phosphorus metabolism biomarkers in the context of OA. Further prospective and longitudinal investigations are required to verify these associations. By clarifying the role of calcium–phosphorus metabolism balance in OA, we aim to offer valuable insights. These insights can then be translated into targeted intervention strategies to alleviate the burden of OA in the United State.

## Acknowledgments

We would like to thank the NHANES for providing the publicly available data used in this study.

## Author contributions

**Conceptualization:** Xiaoyu Gao.

**Data curation:** Huxiong Zhang, Yonghua Wang.

**Formal analysis:** Junjie Kang.

**Methodology:** Tiantian Wang.

**Resources:** Junjie Kang, Keyu Zhao.

**Software:** Junjie Kang.

**Supervision:** Suyalatu Xin.

**Writing – original draft:** Junjie Kang, Yonghua Wang.

**Writing – review & editing:** Junjie Kang, Yonghua Wang.

## Supplementary Material


