# Supplementary material for: Investigating the association between calcium–phosphorus balance and osteoarthritis: Evidence from NHANES 2007–2016
Source: Medicine (Baltimore). 2025 Jul 18;104(29):e43301. doi: 10.1097/MD.0000000000043301 (PMC12282842; doi:10.1097/MD.0000000000043301)
Supplement: Supplementary file 1 [file medi-104-e43301-s001.docx]

**Supplemental Table 1.** Baseline characteristics of individuals aged 30 years and older.

| **Characteristic** | **Overall**  (n = 24338)*^1^* | **Excluded**  (n = 12675)*^1^* | **Included**  (n = 11663)*^1^* | **p***^2^* |
| --- | --- | --- | --- | --- |
| **Age (years)** | 52.63 (14.23) | 55.68 (14.58) | 50.24 (13.48) | <0.001 |
| **Gender (weighted %)** |  |  |  | <0.001 |
| Female | 12,096 (52.36%) | 6,724 (58.47%) | 5,372 (47.56%) |  |
| Male | 11,358 (47.64%) | 5,067 (41.53%) | 6,291 (52.44%) |  |
| **Race (weighted %)** |  |  |  | <0.001 |
| Mexican American | 3,465 (7.69%) | 1,774 (8.15%) | 1,691 (7.33%) |  |
| Other Hispanic | 2,505 (5.24%) | 1,291 (5.70%) | 1,214 (4.89%) |  |
| Non-Hispanic White | 10,059 (68.75%) | 4,741 (64.89%) | 5,318 (71.78%) |  |
| Non-Hispanic Black | 4,933 (10.93%) | 2,770 (13.25%) | 2,163 (9.11%) |  |
| Other/multiracial | 2,492 (7.39%) | 1,215 (8.01%) | 1,277 (6.90%) |  |
| **Education (weighted %)** |  |  |  | <0.001 |
| Less than 9th grade | 2,931 (6.59%) | 1,905 (9.59%) | 1,026 (4.23%) |  |
| 9-11th grade (Includes 12th grade with no diploma) | 3,459 (11.17%) | 2,023 (13.99%) | 1,436 (8.96%) |  |
| High school graduate/GED or equivalent | 5,236 (21.90%) | 2,722 (23.71%) | 2,514 (20.48%) |  |
| Some college or AA degree | 6,318 (29.60%) | 3,018 (29.17%) | 3,300 (29.93%) |  |
| College graduate or above | 5,480 (30.75%) | 2,093 (23.54%) | 3,387 (36.39%) |  |
| (Missing) | 36 | 36 | 0 |  |
| **Body Mass Index (kg/m²)** | 29.28 (6.72) | 30.15 (7.28) | 28.61 (6.17) | <0.001 |
| (Missing) | 1235 | 1235 | 0 |  |
| **Smoking Status (weighted %)** |  |  |  | <0.001 |
| Current Smoker | 4,666 (19.14%) | 2,413 (20.85%) | 2,253 (17.81%) |  |
| Former Smoker | 6,224 (27.25%) | 3,232 (27.93%) | 2,992 (26.71%) |  |
| Never Smoker | 12,543 (53.61%) | 6,125 (51.22%) | 6,418 (55.48%) |  |
| (Missing) | 25 | 25 | 0 |  |
| **Alcohol Consumption** |  |  |  | <0.001 |
| Non-drinker | 6,293 (23.96%) | 3,328 (30.00%) | 2,965 (20.18%) |  |
| <5 drinks/month | 10,241 (49.06%) | 4,459 (48.88%) | 5,782 (49.18%) |  |
| 5-10 drinks/month | 1,445 (8.38%) | 495 (6.38%) | 950 (9.63%) |  |
| >10 drinks/month | 3,105 (18.60%) | 1,139 (14.74%) | 1,966 (21.01%) |  |
| (Missing) | 3254 | 3254 | 0 |  |
| **Diabetes (weighted %)** |  |  |  | <0.001 |
| Diabetes | 4,722 (15.19%) | 2,976 (20.44%) | 1,746 (11.07%) |  |
| Non-Diabetes | 18,732 (84.81%) | 8,815 (79.56%) | 9,917 (88.93%) |  |
| (Missing) | 884 | 884 |  |  |
| **MET (mins/week)** | 4,293.21 (5,978.62) | 4,105.21 (5,857.43) | 4,356.21 (6,017.60) | 0.066 |
| (Missing) | 7379 | 7379 | 0 |  |
| **Calcium (mmol/L)** | 2.35 (0.09) | 2.35 (0.10) | 2.35 (0.09) | 0.330 |
| (Missing) | 2367 | 2367 | 0 |  |
| **Phosphorus (mmol/L)** | 1.21 (0.18) | 1.21 (0.18) | 1.20 (0.18) | 0.006 |
| (Missing) | 2342 | 2342 | 0 |  |
| **Ca/P Ratio** | 1.99 (0.31) | 1.98 (0.31) | 2.00 (0.31) | 0.012 |
| (Missing) | 2371 | 2371 | 0 |  |
| *^1^*Mean (SD); n (unweighted) (%) | | | | |
| *^2^*Design-based t-test; Pearson's X^2: Rao & Scott adjustment | | | | |

**Supplemental Table 2.** GVIF analysis: Calcium levels and related covariates.

| **Variables** | **GVIF** | **DF** | **Adjusted GVIF** |
| --- | --- | --- | --- |
| **Age (years)** | 2.00 | 1 | 1.42 |
| **Gender** | 1.84 | 1 | 1.36 |
| **Education** | 4.39 | 4 | 1.20 |
| **Race** | 3.54 | 4 | 1.17 |
| **BMI** | 1.69 | 1 | 1.30 |
| **Smoking Status** | 4.44 | 2 | 1.45 |
| **Alcohol Consumption** | 3.13 | 3 | 1.21 |
| **MET** | 1.82 | 1 | 1.35 |
| **Diabetes** | 1.80 | 1 | 1.34 |
| **Calcium (mmol/L)** | 1.45 | 1 | 1.20 |

GVIF: Generalized variance inflation factor. DF: Degrees of freedom. Adjusted GVIF: The square root of GVIF corrected by the degrees of freedom.

Model were Adjusted for age, gender, race, education, BMI, smoking status, alcohol consumption, MET and diabetes.

**Supplemental Table 3.** GVIF analysis: Phosphorus levels and related covariates.

| **Variables** | **GVIF** | **DF** | **Adjusted GVIF** |
| --- | --- | --- | --- |
| **Age (years)** | 1.97 | 1 | 1.41 |
| **Gender** | 2.30 | 1 | 1.52 |
| **Education** | 4.50 | 4 | 1.21 |
| **Race** | 3.67 | 4 | 1.18 |
| **BMI** | 1.66 | 1 | 1.29 |
| **Smoking Status** | 4.59 | 2 | 1.46 |
| **Alcohol Consumption** | 3.19 | 3 | 1.21 |
| **MET** | 1.72 | 1 | 1.31 |
| **Diabetes** | 1.94 | 1 | 1.39 |
| **Phosphorus (mmol/L)** | 1.77 | 1 | 1.33 |

GVIF: Generalized variance inflation factor. DF: Degrees of freedom. Adjusted GVIF: The square root of GVIF corrected by the degrees of freedom.

Model were Adjusted for age, gender, race, education, BMI, smoking status, alcohol consumption, MET and diabetes.

**Supplemental Table 4.** GVIF analysis: Ca/P ratio and related covariates.

| **Variables** | **GVIF** | **DF** | **Adjusted GVIF** |
| --- | --- | --- | --- |
| **Age (years)** | 1.98 | 1 | 1.41 |
| **Gender** | 2.27 | 1 | 1.51 |
| **Education** | 4.56 | 4 | 1.20 |
| **Race** | 3.64 | 4 | 1.18 |
| **BMI** | 1.66 | 1 | 1.29 |
| **Smoking Status** | 4.59 | 2 | 1.46 |
| **Alcohol Consumption** | 3.11 | 3 | 1.21 |
| **MET** | 1.71 | 1 | 1.30 |
| **Diabetes** | 1.98 | 1 | 1.41 |
| **Ca/P Ratio** | 1.75 | 1 | 1.32 |

GVIF: Generalized variance inflation factor. DF: Degrees of freedom. Adjusted GVIF: The square root of GVIF corrected by the degrees of freedom.

Model were Adjusted for age, gender, race, education, BMI, smoking status, alcohol consumption, MET and diabetes.
